# Trophic Facilitation or Limitation? Comparative Effects of Pumas and Black Bears on the Scavenger Community

**DOI:** 10.1371/journal.pone.0102257

**Published:** 2014-07-10

**Authors:** Maximilian L. Allen, L. Mark Elbroch, Christopher C. Wilmers, Heiko U. Wittmer

**Affiliations:** 1 School of Biological Sciences, Victoria University of Wellington, Wellington, New Zealand; 2 Panthera, New York, New York, United States of America; 3 Center for Integrated Spatial Research, Environmental Studies Department, University of California Santa Cruz, Santa Cruz, California, United States of America; University of Western Ontario, Canada

## Abstract

Scavenging is a widespread behaviour and an important process influencing food webs and ecological communities. Large carnivores facilitate the movement of energy across trophic levels through the scavenging and decomposition of their killed prey, but competition with large carnivores is also likely to constrain acquisition of carrion by scavengers. We used an experimental approach based on motion-triggered video cameras at black-tailed deer (*Odocoileus hemionus columbianus*) carcasses to measure the comparative influences of two large carnivores in the facilitation and limitation of carrion acquisition by scavengers. We found that pumas (*Puma concolor*) and black bears (*Ursus americanus*) had different effects on their ecological communities. Pumas, as a top-level predator, facilitated the consumption of carrion by scavengers, despite significantly reducing their observed sum feeding times (165.7 min±21.2 SE at puma kills 264.3 min±30.1 SE at control carcasses). In contrast, black bears, as the dominant scavenger in the system, limited consumption of carrion by scavengers as evidenced by the observed reduction of scavenger species richness recorded at carcasses where they were present (mean = 2.33±0.28 SE), compared to where they were absent (mean = 3.28±0.23 SE). Black bears also had large negative effects on scavenger sum feeding times (88.5 min±19.8 SE at carcasses where bears were present, 372.3 min±50.0 SE at carcasses where bears were absent). In addition, we found that pumas and black bears both increased the nestedness (a higher level of order among species present) of the scavenger community. Our results suggest that scavengers have species-specific adaptions to exploit carrion despite large carnivores, and that large carnivores influence the structure and composition of scavenger communities. The interactions between large carnivores and scavengers should be considered in future studies of food webs and ecological communities.

## Introduction

Carrion is an essential but temporal resource for countless species ranging from microbes to vertebrates [1]–[3]. Both the direct and indirect effects of carrion, and the intense competition that occurs over these resources, are increasingly being recognized as important processes in structuring ecological communities [2]–[6]. For example, by transferring energy across trophic levels, scavengers are thought to increase the stability of ecological communities and food webs [1], [2], [6]. In fact, Wilson and Wolkovich [6] reported that in many food webs, a greater amount of energy is transferred through scavenging of carrion than through direct predation. This is at least partly due to the numerous vertebrate species which adopt scavenging to increase reproductive success and survival, and hence their individual fitness (e.g., [7]–[9]). Despite scavenging being a wide-spread and ecologically significant behaviour, our understanding of the biotic constraints that limit access to carrion for vertebrate scavengers is limited [1], [3].

Carcasses of large bodied ungulate species are a particularly rich source of nutrition, especially during lean seasons such as late winter in North America when many species, including carnivores, struggle to meet their energetic requirements [2], [3], [5], [8]. However, ungulate carcasses are also patchily distributed and only available for short periods of time [1], [3], [6]. Competition, one of the most important processes in evolution and ecology [10], among facultative scavenger species is therefore likely to be an important factor in the acquisition of carrion, with the potential to influence community assemblages at larger scales [4], [11], [12].

Many large carnivores facilitate the acquisition of carrion for scavengers and decomposers [2], [4], [13], but large carnivores and other dominant scavengers also compete with and limit the consumption of carrion by smaller species [2], [12], [14]–[16]. Instances of ‘trophic facilitation’ occur when scavengers from different trophic levels benefit from the leftover carrion from carnivore kills without negatively influencing the carnivore that made the kill [2], [16]. Instances of ‘trophic limitation’ occur when predators or dominant scavengers monopolize carrion resources and in turn limit access of subordinate scavenger species. Through trophic facilitation and limitation the potential exists for large carnivores to influence the presence and behaviours of scavengers at carrion resources, as well as potentially influence the structure and composition of the scavenger community. Recent studies have shown resource partitioning, specific niches, and nested relationships within the scavenger community [5], [17], [18]. Nested relationships (nestedness is an analysis of presence and absence used to measure order and disorder in patterns of species occurrence [19]) suggest that the diversity of scavengers present in a location is governed by specific circumstances as well as complex processes and relationships.

Pumas (*Puma concolor*) and black bears (*Ursus americanus*) are sympatric large carnivores occurring across much of North America but with different ecological niches. Pumas are top-level predators that frequently kill ungulates [20], while bear species are dominant facultative scavengers [21]. Scavengers frequently feed at puma kills [13], [20], [22], [23], suggesting that pumas facilitate the acquisition of carrion by scavengers, and may in turn play a keystone role by supporting a diverse scavenger community [13]. In contrast, black bears rarely kill adult ungulates, and instead opportunistically feed on juvenile ungulates or carcasses of adults when available [24]–[26]. Black bears are also able to usurp kills from other large carnivores [20], [21], [27]. This suggests that black bears may be a dominant scavenger that has a competitive advantage in the consumption of carrion over other scavenger species.

We attempted to determine the influence of pumas and black bears on species richness, feeding time, and nestedness of the scavenger community at ungulate carcasses. To achieve our objective, we conducted a series of *in situ* experiments at black-tailed deer (*Odocoileus hemionus columbianus*) carcasses which we monitored with motion-triggered video cameras. We first determined the influence of pumas on scavengers by comparing species richness, feeding time, and nestedness at deer killed by pumas wearing GPS collars to control carcasses with matched habitat characteristics. Second, we determined the influence of black bears on scavengers, using a separate set of carcasses distributed by researchers, by comparing the variables at carcasses where black bears were present and absent. This design allowed us to compare the influence of large carnivores on three aspects of scavenger ecology: 1) scavenger species richness (the number of scavenger species at each carcass), 2) scavenger sum feeding times (the total time scavengers spent at each carcass as a proxy for energy gained), and 3) the nestedness of the scavenger community at these carcasses. We also examined how the first two of these variables varied seasonally. We hypothesized that pumas and black bears would both limit scavenger species richness and sum feeding times, while also increasing the nestedness of the scavenger community. This contrasts with previous studies about the effect of dominant scavengers, which found that dominant scavengers do not influence scavenger species richness or the nestedness of the scavenger community (e.g., [28], [29]). However, as a large carnivore we expected black bears would have larger effects on the scavenger community than avian scavengers or mesocarnivores. Further, we expected the effects of pumas and black bears to be of a similar magnitude, but that the net effect of pumas and black bears as sources of trophic facilitation and limitation for the scavenger community would vary based on their different ecological roles as a top predator and dominant scavenger.

## Methods

Our protocols for the capture of pumas adhered to the guidelines outlined by the American Society of Mammalogists [30], and were approved by the Institutional Animal Care and Use Committee of the University of California, Davis (Protocols 15341 and 16886), and the Wildlife Investigations Lab of the California Department of Fish and Wildlife. Every effort to ameliorate suffering of cougar subjects was made, and no cougars were ever killed/sacrificed as part of research methods. Our research was carried out on the Mendocino National Forest and adjacent private land. No permits are necessary for conducting research on Mendocino National Forest, and permission to use private land was granted by T. McIsaac and B. Hurt. Pumas are neither threatened nor endangered, and permission to handle pumas was granted through a Memorandum of Understanding with the California Department of Fish and Wildlife.

### Study Area

**Figure 1:**
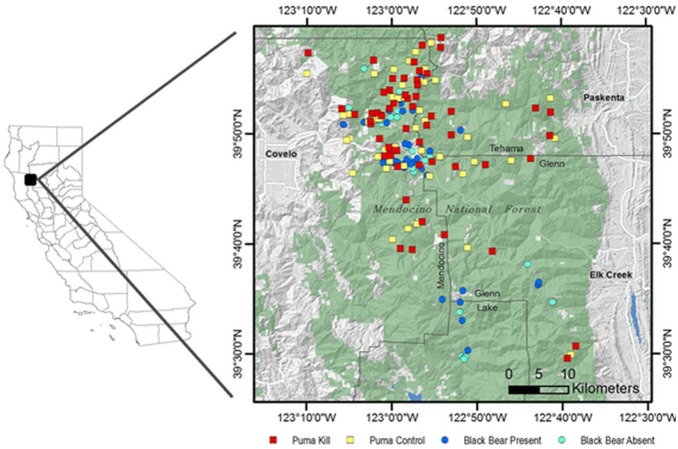
A map of the study area, in Mendocino National Forest. The study area is outlined by the thick black line, within the greater context of the North Coast Range and California.

We conducted our study in the Mendocino National Forest, California, including portions of Mendocino, Tehama, Glenn, and Lake Counties (Figure 1). Our study area encompassed approximately 1,000 km^2^, with elevations from 396 to 2,466 m. Mean daily temperatures ranged from −1°C to 24°C, and mean annual precipitation averaged 132 cm. The majority of precipitation occurred from December through March; below 1,000 m precipitation was frequently in the form of rain, while at higher elevations precipitation was more frequently snow.

Major habitat types changed with elevation. Based on the California Wildlife Habitat Relationships categories [31] these included (in order of increasing elevation): blue oak (*Quercus douglasii*) woodland, annual grassland, montane hardwood conifer, Douglas fir (*Psuedotsuga menziesii*), mixed chaparral, montane hardwood, ponderosa pine (*Pinus ponderosa*), Klamath mixed conifer, and montane chaparral. Black-tailed deer were the most common large ungulate prey, and pumas are the only predator in the system which frequently killed adult deer [32]. Wild pigs (*Sus scrofa*) and tule elk (*Cervus elaphus nannodes*) were present at low densities in the study area, but were not preyed upon by pumas [32]. Pumas in the study area occurred at comparatively low density (2.45/402 km^2^), while occurrence of black bears was high and evenly distributed [33].

### Experimental Design and Field Methods

We employed motion-triggered video cameras with infrared flash at black-tailed deer carcasses to measure the effects of pumas and black bears on scavenger activity (species richness, sum feeding times, and nestedness of occurrence). To test for effects of pumas, we compared scavenger activity at kills made by pumas (n = 58; n_winter_ = 10, n_spring_ = 11, n_summer_ = 16, n_autumn_ = 21) to control carcasses that we placed simultaneously on the landscape in areas with matching habitat characteristics (n = 58; n_winter_ = 10, n_spring_ = 11, n_summer_ = 16, n_autumn_ = 21). In the puma experiments, black bears were considered a scavenger. To test for the effects of black bears, we distributed a different set of black-tailed deer carcasses in different habitat types across the study area, and determined their effects by comparing scavenger activity at carcasses where black bears were present (n = 43; n_winter_ = 1, n_spring_ = 15, n_summer_ = 15, n_autumn_ = 12) to carcasses where they were absent (n = 47; n_winter_ = 25, n_spring_ = 20, n_summer_ = 1, n_autumn_ = 1).

We used black-tailed deer killed through vehicle collisions for puma control carcasses and all of the carcasses used for the black bear experiments. Deer were collected from highways in Mendocino, Lake, Glenn, Sonoma, and Marin Counties. We only collected fresh deer in good condition allowing us to replicate the decomposition states of deer at puma kills. Each deer collected had clear, unclouded eyes, lacked discoloration in the abdominal region, and lacked obvious external wounds (broken bones, punctures of skin, or abrasions). Before transporting the carcasses we removed their head, entrails, and lungs in order to limit any disease transmission among disjunct deer populations, as required by the California Department of Fish and Wildlife. In many systems smaller scavengers are dependent on larger scavengers to open carcasses and allow them access to meat (e.g., [34]). Given carcass handling described above, small scavengers were not dependent on other species for feeding.

Between June 2010 and December 2012, we captured 7 pumas and fitted them with a combined ARGOS satellite GPS/radio telemetry collar (Lotek 7000SAW, New Market, Ontario), using the methods described in Allen [32]. In order to find fresh deer killed by pumas, we programmed the collars to acquire GPS locations at 2-hr intervals throughout each 24-hr period, and downloaded the location data via satellite connection every 3 days. Location data were displayed in ArcGIS 3.2, and we defined GPS clusters as ≥2 locations within 150 m of each other that contained at least one crepuscular or nocturnal location [35]. We loaded GPS clusters onto handheld GPS units (Garmin 60csx), and investigated any GPS cluster where the puma appeared to still be present on the same day as the Argos-relayed GPS downloads in order to find puma kills while they were fresh and the pumas were still active at the site.

Upon arrival in the vicinity of the GPS cluster, we listened for the puma’s presence with a handheld telemetry receiver (Communication Specialists Model R1000, Orange, CA). If the puma was in the immediate vicinity we considered it active at the feeding site. We then approached the potential feeding site during mid-day when pumas are least active. We limited visits to 5 min at the site in order to reduce disturbance and avoid possible carcass abandonment. When we found at least half of the deer still left we used the site for our experiments. We attached a wire cable to the carcass to hold it within 1 m of its location, and deployed a motion-triggered camera (Bushnell TrophyCam, Overland Park, KS) to monitor the carcass and document scavenger activity. The motion-triggered cameras were set to record the maximum amount of activity: 60 s of video and inter-video lengths of 1 s before the next event was recorded. We then recorded primary and secondary habitat characteristics of the site based on Mayer and Laundenslayer [31]. We also recorded the location and elevation of the kill using a handheld GPS unit, and then estimated the distance to the secondary habitat by pacing off the distance. Finally, we measured the percent canopy cover directly over the carcass with a spherical concave densiometer (Ben Meadows, Janesville, WI), and the tree species with the highest percentage of overhead cover.

In finding potential matching sites for puma control carcasses we attempted to choose habitat characteristics as closely as possible to the following variables at puma kills (listed in order of importance): 1) primary habitat type, 2) overhead tree species, 3) percent canopy cover, 4) secondary habitat type, 5) distance to secondary habitat, 6) elevation (Appendix S1). Paired carcasses were placed at least 1 km from puma kills to reduce detection by the same individual scavengers, and each carcass site was used only once to avoid conditioning scavengers. Before arriving at the control sites, we prepared road-killed black-tailed deer to match the amount of meat and stage of decomposition of carcasses found at puma kills. Puma control carcasses were prepared and deployed soon after we visited the puma-killed carcass (a mean of 1.51 hours±0.18 SE) to rule out possible effects from weather, and were also secured with a wire cable.

For the black bear experiments we placed 100 black-tailed deer carcasses in the study area from December 2009–October 2012. Carcasses were placed in a variety of habitat conditions (Appendix S2) in order to provide a diverse sample of the vertebrate scavenger community. Habitat conditions measured included primary habitat type, secondary habitat type, the distance to secondary habitat, overhead tree species, percent canopy cover, slope, aspect, and elevation. We anchored each carcass in place with a wire cable, and placed a motion-triggered camera (Cuddeback IR, De Pere, WI) on a nearby tree. The motion-triggered cameras were set to record the maximum amount of activity, with 30 s of video with a pause of 60 s before the next trigger.

We then determined the effects of black bears by comparing scavenger activity at carcasses where black bears were present to carcasses where they were absent. We included instances where a black bear first arrived at a carcass in its last stage of consumption where scavengers were feeding on the leftover hide and bones (e.g., [2]) in the absent class (defined as where they were absent or one of the last scavengers to arrive and spent ≤10 min at the carcass). We removed carcasses from our analyses which had incomplete data due to camera malfunctions or camera displacements by black bears (n = 10).

### Statistical Analyses

We determined the number of scavenger species at each carcass and the amount of time they spent feeding to the closest min using the videos we recorded. We calculated the duration of each feeding bout by a scavenger species by subtracting the time at the start of a visit by the time at the end of a visit, with multiple individuals of a species all combined in one feeding bout. For visits of <30 s we considered the species present for 1 min rather than 0 min, and we rounded all other visits to the closest min. Total feeding times were calculated as the sum of all feeding bouts for all scavengers at a given carcass. We then performed analyses to determine the effects of pumas and black bears on scavenger species richness, scavenger sum feeding times, and the nestedness of the observed scavenger communities. Each analysis determined the influence of either pumas (kills vs. control carcasses) or black bears (present vs. absent). There was, however, limited utility in comparing the effects of black bears to pumas directly due to the varying amounts of meat, the different camera models used, and how these variables could affect scavenger presence and feeding times. Therefore, we used post hoc effect sizes [36] to compare the effects of pumas and black bears on scavengers. Prior to performing statistical analyses we tested each data set with continuous variables for normality and variance equality with a Shapiro-Wilk test and a Levene’s test [37]. In each analysis, we considered *p*≤0.05 significant, and the statistical analyses were conducted using the program R [38], except where specifically noted otherwise.

We first tested whether black bear detection of carcasses would vary among seasons because of expected variation in abundance among seasons due to hibernation using a Fisher’s exact test [37]. For this analysis, and the later analyses which included the predictor variable of season, we assigned seasons based on ecological patterns in the study area. Winter included December, January, and February; spring included March, April, May; summer included June, July, and August; and autumn included September, October, November [32].

For species richness we used all scavenger species, but eliminated rodents and small birds (i.e. American robin, *Turdus migratorius*, scrub jay, *Aphelocoma californica*, and Steller’s jay, *Cyanocitta stelleri*) for sum feeding times due to limitations in our ability to accurately detect their feeding times. We modelled scavenger species richness and scavenger sum feeding times using generalized linear models with a Poisson link, using the *lme4* package [39]. We first modelled the puma experimental carcasses, using puma carcass type (kill vs. control), season, and their interaction as dependent variables. We then modelled the black bear experimental carcasses, using black bear carcass type (bear present vs. absent), season, and their interaction as dependent variables. When significant differences were found we used a post hoc Tukey’s HSD test to determine where significant differences occurred. Last, we calculated post hoc effect sizes using Cohen’s *d* score for scavenger species richness and sum feeding times in order to understand the magnitude of effects, and we considered scores of 0.20 small effects, 0.50 medium effects, and 0.80 large effects [40].

Our hypothesized limitation of scavenger species richness and sum feeding times could cause an increase in the order or disorder of the scavengers present at different carcass types. For instance, competition with a puma or black bear could cause the species which are present and able to feed to be more random. In contrast, if specific scavengers have developed strategies to overcome competitive restraints from pumas and black bears this would cause their presence at a particular carcass to be more structured. Based on Selva and Fortuna [5], we therefore hypothesized that the scavenger community of each carcass type would be more nested than random null models. In addition, as noted previously, we hypothesized that the scavenger community would be more nested at puma kills than control carcasses, while the nestedness of carcasses where black bears were present vs. absent would not vary significantly. To test this, we used a nestedness analysis following the methods which Selva and Fortuna [5] used for a vertebrate scavenger community in Europe. An analogy often used to explain nestedness is the species occurrence among a series of same-size islands moving away from the mainland. In a system which is nested due to dispersal from the mainland, the species are structured by their distance from the mainland. If instead the island system were based on a random process, for example with species being distributed during tropical storms, the species present on each island would be less structured and hence less nested.

We calculated the nestedness temperature (*T*) [19], [41] for each carcass type using the program ANINHADO (16), with *T* expressed as a score between 0–1. We then calculated the level of nestedness (*N*) as used by Selva and Fortuna [5] who defined *N* as *N* = (100–*T*)/100 for each carcass type. We also calculated the idiosyncratic temperature (*IT*) first for each individual carcass and second for each scavenger species. We then calculated the nestedness contribution (*NC*) for each individual carcass and each scavenger species as *NC* = (100–*IT*)/100 [5]. *T* is the mean value of the *IT* scores of all individual carcasses, or alternatively the mean of all individual scavengers since both sides of the matrix have the same mean, and hence *N* is the mean value of either of their *NC* scores. Higher N and NC scores meant the scavenger community or species was structured and hence nested, while low scores meant less structure and hence disordered.

We determined if each carcass type was significantly more nested than random by comparing each to random null models. For each carcass type we generated 100 null models with randomized matrices for each of two null model types (null model 1 lacking heterogeneity and nestedness, and null model 2 lacking nestedness) using the program ANINHADO [19]. We then tested the *N* score for each type of carcass against both types of their randomly generated null models using ANOVA models [37], and when we found significant differences we used a post hoc Tukey’s HSD test [37] to determine where the significant differences occurred. Lastly, we determined if pumas and black bears increased or decreased the nestedness, and hence the structure, of the scavenger community. We used the individual *NC* scores from each individual carcass as our values, and used a two-tailed Student’s t-test with equal variances [37] determine differences in *N* caused by pumas and black bears.

## Results

**Figure 2:**
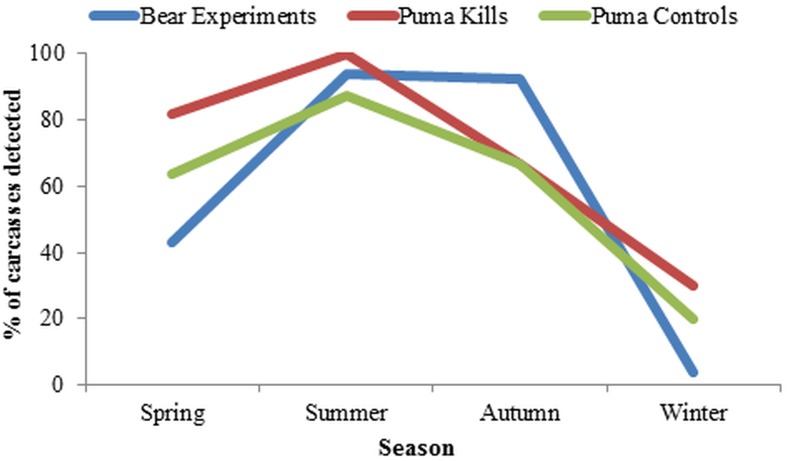
The detection rate of black bears among seasons at different carcass types. Detection varied among seasons, and the detection for deer carcasses used for the black bear experiments, those killed by pumas, and the matching puma control carcasses are shown.

We monitored 58 puma kills and 58 puma control carcasses. We set up cameras at the puma kills a mean of 39.8 (±2.9 SE) hours after the presumed time of kill, and pumas stayed within 150 m of the kills we monitored for a mean of 75.1 (±5.9 SE) hours. For the black bear experiments we monitored 47 carcasses where black bears were absent and 43 carcasses where black bears were present. Black bear occurrence at carcasses varied by season (df = 3, *p*<0.0001, Figure 2), as would be expected based on their seasonal activity patterns. Black bears were present at 92.8% and 90.0% of experimental carcasses during summer and autumn respectively, compared to 48.6% in spring and 3.8% in winter.

### Species richness

We recorded a total of 20 species (Appendix S1) at puma kills with a mean of 3.07 (±0.24 *SE*) species per carcass, and a total of 25 species (Appendix S1) at paired control carcasses with a mean of 3.52 (±0.20 *SE*) species per carcass, resulting in a small negative effect attributable to pumas (*d* = −0.27) (Table 1). Neither puma carcass type (kill vs. control), season, nor the interaction between season and carcass type was a significant factor in scavenger species richness (Table 2). Puma carcass type was the model with the lowest AIC value to explain scavenger richness (Table 2).

**Table 1:** The mean scavenger species richness and scavenger sum feeding time, along with the effect size attributable to pumas and black bears.

	Scavenger Richness			Sum Feeding Time		
Variables	mean	95% CI	*d*	mean	95% CI	*d*
Puma Kill	3.07	2.60–3.54	−0.27	165.7	124.1–207.3	−0.41
Puma Control	3.52	3.13–3.90		246.3	187.3–305.3	
Bear Present	2.33	2.06–2.62	−0.56	88.5	68.7–108.3	−1.08
Bear Absent	3.28	3.05–3.51		372.3	322.3–422.3	

For each variable and carcass type the mean, 95% confidence intervals, and effect sizes (Cohen’s *d* score) are reported. Negative effect sizes indicate limitation, with effect sizes of 0.20 indicating small effects, 0.50 indicating medium effects, and 0.80 indicating large effects.

**Table 2:** The results modelled for the interaction of season with scavenger species richness and sum feeding time.

		Scavenger Richness				Sum Feeding Time			
Model	Variables	df	*F*	*p*	AIC	df	*F*	*p*	AIC
1	PUMA	1	2.12	0.1499	441.0	1	4.96	0.0280	201.8
2	SEAS	3	1.28	0.2860	443.6	3	1.65	0.1820	202.1
3	PUMA:SEAS	3	0.99	0.3982	447.5	3	1.61	0.1910	185.5
1	BEAR	1	7.01	0.0096	344.3	1	26.04	<0.0001	203.7
2	SEAS	3	5.02	0.0030	339.1	3	3.91	0.0114	240.7
3	BEAR:SEAS	3	2.20	0.0940	341.0	3	0.69	0.5631	193.9

The variables include pumas (puma kill vs. control), black bears (black bear present vs. absent), and the 4 seasons. The statistical significance and Akaike’s Information Criterion of each model is reported.

Model variables: PUMA = puma carcass type (puma kill or puma control carcass), SEAS = season of year (winter, spring, summer, or autumn), BEAR = black bear carcass type (carcasses where black bear is present or carcass where black bear is absent).

**Figure 3:**
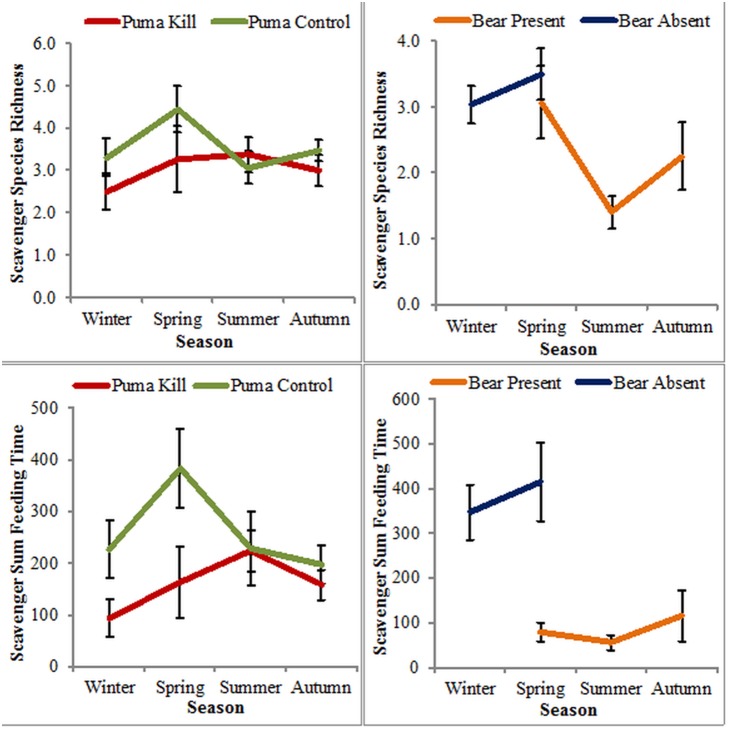
The seasonal distribution of scavenger species richness (number of scavengers present) and sum feeding time in minutes (total time scavengers spent feeding). Each season is represented as a mean with the error bars representing standard error. We did not include samples on the graphs where there were <3 samples for a season.

We recorded a total of 21 species (Appendix S2) at carcasses where black bears were present with a mean of 2.33 (±0.28 SE) species per carcass, and a total of 18 species (Appendix S2) at carcasses where black bears were absent with a mean of 3.28 (±0.23 SE) species per carcass, resulting in a medium negative effect attributable to black bears (*d* = −0.56) (Table 1). Black bear carcass type (present vs. absent) was a significant factor in scavenger species richness (df = 1, *F* = 7.01, *p* = 0.0096, Table 2). Season alone was also a significant factor in scavenger species richness at black bear carcasses (df = 3, *F* = 5.02, *p* = 0.0030, Table 2), with significantly less species per carcass in summer (mean = 1.50±0.24 SE) than in spring (mean = 3.31±0.32 SE, *p* = 0.0025) or winter (mean = 3.15±0.30 SE, *p* = 0.0117). The interaction of black bear carcass type and season was not significant (Table 2, Figure 3). Season was the model with the lowest AIC value to explain scavenger richness (Table 2).

### Scavenger Sum Feeding Times

At puma kills scavengers fed for a mean of 165.7 (±21.2 SE) min, and at puma control carcasses scavengers fed for a mean of 246.3 (±30.1 SE) min, resulting in a medium negative effect attributable to pumas (d = −0.41) (Table 1). Puma carcass type (kill vs. control) was a significant factor in scavenger sum feeding times (df = 1, *F* = 4.96, *p* = 0.0280, Table 2). Season alone was not a significant factor of scavenger sum feeding times (Table 2), nor was the interaction between puma carcass type and season significant (Table 2), but based on AIC values was the best model to explain scavenger sum feeding times (Table 2).

At carcasses where black bears were present scavengers fed for a mean of 88.5 (±19.8 SE) min, while at carcasses where bears were absent scavengers fed for a mean of 372.3 (±50.0 SE) min, resulting in a large negative effect attributable to black bears (*d* = −1.08) (Table 1). Black bear carcass type (present vs. absent) was a significant factor in scavenger sum feeding times (df = 1, *F* = 26.04, *p*<0.0001, Table 2). Season alone was also a significant factor in scavenger sum feeding times (df = 3, *F* = 3.91, *p* = 0.0114, Table 2), with significantly higher sum feeding times in winter (mean = 347.6 min±60.0 SE) than in summer (mean = 72.6 min±23.8 SE, *p* = 0.0162). The interaction between black bear carcass type and season was not significant, but ranked the best model to explain scavenger sum feeding times (Table 2).

### Nestedness of scavenger interactions with pumas and black bears

Our analyses revealed that each carcass type (puma kill, puma control, black bear present, black bear absent) was significantly more nested than the randomly generated null models (Table 3); showing structured and nested relationships among the scavenger community. The randomly generated null models of type 1 lacked heterogeneity and nestedness, and were significantly lower than each carcass type (*p_puma kill_*<0.0001, *p_puma control_*<0.0001, *p_bear present_*<0.0001, *p_bear absent_*<0.0001). The randomly generated null models of type 2 lacked nestedness, and were significantly lower than each carcass type (*p_puma kill_*<0.0001, *p_puma control_* = 0.0012, *p_bear present_*<0.0001, *p_bear absent_*<0.0001).

**Table 3:** Comparison of the nestedness (*N*) scores of each carcass type with two types of randomly generated null models.

		ANOVA			Null Model 1		Null Model 2	
Carcass Type	*N (SE)*	*df*	*F*	*p*	*N* (*SE*)	*p*	*N* (*SE*)	*p*
Puma Kills	0.88 (0.01)	2, 198	250.46	<0.0001	0.55 (0.004)	<0.0001	0.67 (0.004)	<0.0001
Puma Control	0.83 (0.02)	2, 198	206.23	<0.0001	0.58 (0.004)	<0.0001	0.69 (0.004)	0.0012
Black Bear Present	0.92 (0.02)	2, 198	298.98	<0.0001	0.64 (0.004)	<0.0001	0.73 (0.005)	<0.0001
Black Bear Absent	0.83 (0.02)	2, 198	241.96	<0.0001	0.53 (0.005)	<0.0001	0.64 (0.005)	<0.0001

Null models of type 1 lacked heterogeneity and nestedness, and while null models of type 2 lacked nestedness. Nestedness (*N*) is represented for each carcass type, along with the results of the ANOVA analyses, and the results from post hoc Tukey’s HSD analyses. The mean nestedness (*N*) and the standard error are represented for null model types.

Further analyses revealed that both pumas and black bears increased the nestedness (*N*), and hence structure, of the scavenger community. The scavenger community at puma kills were significantly more nested than puma control carcasses (df = 113, *p* = 0.0468). Nestedness amounted to *N* = 0.88 (±0.01 SE) at puma kills, and *N* = 0.83 (±0.02 SE) at puma control carcasses, indicative of a medium effect attributable to pumas (*d* = 0.38). The scavenger community was significantly more nested at carcasses where black bears were present than carcasses where black bears were absent (df = 84, *p* = 0.0022). Nestedness amounted to *N* = 0.92 (±0.02 SE) at carcasses where black bears were present, and *N* = 0.83 (±0.02 SE) at carcasses where black bears were absent, indicative of a large effect attributable to black bears (*d* = 0.68).

The nestedness contribution (*NC*) scores of scavengers showed apparent differences between carcass types (puma kill vs. control, or black bear present vs. absent), with the NC scores for the most common scavenger species noted in Table 4. Four of these species contributed more to the nestedness of the scavenger community at puma kills, including bobcats (*Lynx rufus*), fishers (*Martes pennanti*), gray foxes (*Urocyon cinereoargenteus*), and turkey vultures (*Cathartes aura*). Five of these species contributed more to the nestedness of the scavenger community at carcasses where black bears were present, including bobcat, common ravens (*Corvus corvax*), coyotes (*Canis latrans*), fishers, and turkey vultures.

**Table 4:** The nestedness contribution (*NC*) scores and occurrence rate for the 7 most frequent scavenger species at each carcass type.

	Puma Kill	Puma Control	Black Bear Present	Black Bear Absent
Species	*NC* (occurrence)	*NC* (occurrence)	*NC* (occurrence)	*NC* (occurrence)
Bobcat	0.91 (8.6%)	0.77 (15.5%)	0.74 (4.7%)	0.54 (27.7%)
Common Raven	0.77 (22.4%)	0.78 (27.6%)	0.90 (41.9%)	0.68 (44.7%)
Coyote	0.75 (25.9%)	0.81 (36.2%)	0.97 (23.3%)	0.64 (38.3%)
Fisher	0.89 (17.2%)	0.53 (22.4%)	0.89 (20.9%)	0.71 (34.0%)
Gray Fox	0.93 (37.9%)	0.85 (48.3%)	0.82 (25.6%)	0.93 (59.6%)
Spotted Skunk	0.83 (17.2%)	0.86 (13.8%)	0.87 (2.3%)	0.92 (10.6%)
Turkey Vulture	0.71 (19.0%)	0.65 (22.4%)	0.94 (46.5%)	0.40 (23.4%)

A higher NC score indicates that the occurrence of the species is more structured, and rate of occurrence is noted as the percent of carcasses at which the species was present.

## Discussion

A driving force for the evolution of carnivores is the adaptations needed to overcome continually evolving defence strategies of prey species [10]. Competitive interactions among vertebrate scavengers may be as complex as predator-prey relationships (e.g., [5], [16]), and should also be the subject of co-evolutionary adaptations and strategies among carnivores and their associated scavenger community. Our results suggest that large carnivores, when acting as either a top predator or dominant scavenger, influence the structure of the scavenger community by both facilitating and limiting the acquisition of carrion by different scavenger species. Considering the importance of interspecific interactions in the acquisition of carrion, large carnivores may be an important cause of adaptation for many vertebrate scavenger species.

The nestedness, or structure of the scavenger community, increased at carcasses where pumas and black bears were present. The scavenger community at each carcass type (puma kill, puma control, bear present, bear absent) was more nested than randomly generated null models, supporting findings of previous studies [5]. But more importantly, our findings suggest species-specific responses to large carnivores (both positive and negative), and that large carnivores are an important influence on the structure and composition of the vertebrate scavenger community whether they are acting as a predator or scavenger. The increased nestedness is likely due to a combination of two factors: 1) competition with pumas and black bears structures the exploitation of carcasses by different species [29], and 2) scavengers with increased nestedness have evolved behaviours and strategies to allow them to take advantage of carrion despite large carnivores. We hypothesize these behaviours are adaptations to increase the predictability of carrion resources, which are generally uncertain and temporal [1], [3], [6]. Increased predictability of carrion could allow scavengers to acquire and exploit the limited available carrion resources, and strategically increase their individual fitness.

The magnitude of the effect of pumas and black bears varied for the species richness and sum feeding times of scavengers. Black bears had a medium effect on scavenger species richness (*d* = −0.56) and a large effect on sum feeding times (*d* = −1.20), while pumas did not significantly limit scavenger species richness, and had a medium negative effect on sum feeding times (*d* = −0.41). The confounding effect of black bears being present at both puma kills and puma control carcasses may have led to an underestimation of the size of the effect attributable to pumas. Pumas also acted as an important facilitator of energy to scavengers in ecosystems. For example, 20 vertebrate scavenger species fed at puma kills, and these scavengers fed for a mean of 165.7 min at each kill. This suggests that pumas provide resources for a large number of scavengers, and may act as a keystone species subsidizing the scavenger community [13], [23]. Furthermore, pumas may increase ecosystem stability by facilitating the movement of energy to different trophic levels [1], [6]. In contrast, black bears are a dominant scavenger, which compete for carrion resources and rarely provide energy to other scavengers (e.g., by killing adult ungulates on their own) [26]. Previous studies of dominant scavengers suggest that they do not limit the richness of other scavenger species [28], [29], or influence the nestedness of the scavenger community [29]. However, black bears exhibited limitations of a higher magnitude for the scavenger community than pumas did. The negative effects of black bears on scavenger species richness and sum feeding times suggest a decrease in the complementary use of carrion by other scavengers in the community, and therefore the effects of black bears may be best classified as trophic limitation. Although both large carnivores limited sum feeding times, and hence could have direct effects on the individual fitness and populations of scavengers, their overall effects are likely to be dependent upon their respective ecological roles.

The seasonal effects of pumas and black bears varied, which is likely due to the variation in seasonal abundance of black bears as explained by hibernation. During seasons they were not hibernating, black bears directly decreased the sum feeding times and species richness at carcasses where they were present, apparently decreasing the amount of carrion available for other species. Black bears may also have influenced the scavenger community at puma kills, as sum feeding times at puma kills and control carcasses did not vary in summer and autumn, suggesting that black bears may have competitive advantages over other scavengers for the available resources at both types of carcasses. Our results therefore suggest that pumas may act as a resource buffer for scavengers, by killing ungulates and facilitating scavengers with carrion resources throughout the year [2]. However, black bears may dampen the beneficial effect of the year-round buffer provided by pumas, and make carrion less available to other scavengers in seasons when they are active. The combined influences of seasonality and predictability of carrion availability are important factors to consider in the evolution of scavenger ecology. Past studies, using two season models, have shown that facultative scavengers scavenge more frequently during winter than summer [9], [16], [21]. However, our study suggests that this may be less due to changes in their preferences or metabolic needs, and instead due to competition with bears and obligative scavengers like vultures, which are less abundant in winter.

In summary, results from our study suggest that large carnivores exert important influences on vertebrate scavengers, which may have implications for community assemblages at larger scales. Pumas apparently facilitate the acquisition of carrion by the scavenger community through the use of carrion from their kills. This suggests that pumas may be provisioning the scavenger community with carrion, and the large amount of carrion provided to scavengers suggest that pumas may be a keystone species for the scavenger community. In contrast, black bears are dominant scavengers, and carrion is apparently an important source of nutrition for them. Black bears were a large source of limitation for the scavenger community, which may have important consequences for the rest of the scavenger community. However, black bears vary in abundance due to hibernation, and competition with black bears during seasons they are active may influence scavenger survival and population dynamics. Large carnivores apparently have different influences based on their ecological role, and also may cause the energy available from carrion resources to be partitioned by specific species or across trophic levels [3]. Our results suggest that the influences of large carnivores on the scavenger community should be considered in future studies of food webs and species interactions within ecological communities.

## Supporting Information

Appendix S1 The habitat characteristics, sum feeding time, and scavengers present at the puma experimental carcasses.
(DOCX)

Appendix S2 The habitat characteristics, sum feeding time, and scavengers present at the black bear experimental carcasses.
(DOCX)
