# Supplementary material for: Trophic Facilitation or Limitation? Comparative Effects of Pumas and Black Bears on the Scavenger Community
Source: PLoS One. 2014 Jul 10;9(7):e102257. doi: 10.1371/journal.pone.0102257 (PMC4092109; doi:10.1371/journal.pone.0102257)
Supplement: Appendix S1 — The habitat characteristics, sum feeding time, and scavengers present at the puma experimental carcasses. (DOCX) [file pone.0102257.s001.docx]

Appendix A. The habitat characteristics, sum feeding time, and scavengers present at the puma experimental carcasses. Appendix A1 is the information for the puma kills, while Appendix A2 is for the puma control carcasses. Habitat characteristics reported include the primary and secondary habitat types [26], the distance to the secondary habitat, the elevation, canopy cover, slope, aspect, and overhead tree species. The scavenger sum feeding times (excluding rodents and small birds), and each of the scavengers that occurred at the carcass are also reported.

A1

| Kill Carcass Name | Primary Habitat | Secondary Habitat | Distance (meters) | Elevation (meters) | Canopy Cover | Slope | Aspect | Overhead Tree | Sum Feeding Time | Scavengers Present |
| --- | --- | --- | --- | --- | --- | --- | --- | --- | --- | --- |
| K_F1_052011 | MCH | CRC | 16 | 1192 | 100% | 18 | 228 | Scrub Oak | 187 | Black bear, common raven, gray fox, white-footed woodrat |
| K_M36_052211 | MHW | AG | 20 | 853 | 97% | 3 | 265 | Live Oak | 39 | Black bear, gray fox, mountain lion |
| K_M36_052611 | KMC | PPN | 42 | 994 | 100% | 16 | 64 | Black Oak | 0 |  |
| K_M36_060511 | DFR | KMC | 2 | 1310 | 100% |  |  | Douglas Fir | 584 | Black bear, common raven, coyote |
| K_M36_061111 | MHW | PPN | 14 | 1572 | 100% | 34 | 104 | Live Oak | 184 | Black bear, California ground squirrel, common raven, white-footed woodrat |
| K_F1_062111 | KMC | MHC | 15 | 1656 | 85% | 22 | 48 | Red Fir | 476 | Black bear, common raven, turkey vulture, white-footed woodrat |
| K_F1_063011 | MHC | MCP | 1 | 1308 | 100% | 3 | 206 | Black Oak | 23 | Black bear, gray fox |
| K_F1_070311 | MHC | AG | 3 | 1202 | 95% | 6 | 88 | Black Oak | 185 | Black bear, coyote |
| K_F17_082811 | KMC | PPN | 10 | 1413 | 86% | 10 | 344 | Ponderosa Pine | 133 | Black bear, coyote, turkey vulture |
| K_M33_091711 | MHC | KMC | 6 | 1277 | 100% | 9 | 198 | Douglas Fir | 29 | Black bear, California ground squirrel, ringtail, western spotted skunk |
| K_F23_091711 | KMC | MHC | 12 | 1332 | 100% | 18 | 51 | Incense Cedar | 192 | Black bear, California ground squirrel, western spotted skunk |
| K_F17_100311 | MHC | KMC | 6 | 1677 | 96% | 20 | 344 | Ponderosa Pine | 30 | common raven, Douglas squirrel |
| K_M33_100911 | KMC | MCP | 8 | 1681 | 100% | 14 | 141 | White Fir | 121 | Black bear |
| K_F19_101611 | MHW | MCH | 20 | 897 | 97% | 16 | 48 | Black Oak | 291 | gray fox, fisher, ringtail, western spotted skunk |
| K_F17_102011 | MHC | AG | 8 | 1354 | 79% | 4 | 263 | Brewer's Oak | 355 | Black bear, California ground squirrel, coyote, deer mouse, western spotted skunk |
| K_F19_102511 | MCH | MHW | 4 | 1193 | 100% | 19 | 226 | Manzanita | 538 | Black bear |
| K_F23_102811 | MHW | MHC | 4 | 1103 | 100% | 18 | 187 | Live Oak | 251 | Black bear, fisher |
| K_F19_110611 | MCH | AG | 25 | 1081 | 100% | 24 | 262 | Scrub Oak | 8 | Bobcat, western spotted skunk, white-footed woodrat |
| K_F17_110911 | MHW | MCP | 2 | 919 | 93% | 28 | 251 | Black Oak | 104 | Gray fox, western spotted skunk |
| K_F23_111111 | MRI | BOP | 35 | 585 | 97% | 17 | 23 | Dogwood | 104 | Black bear, deer mouse, gray fox, ringtail |
| K_F17_112011 | MHC | MHW | 12 | 913 | 93% | 16 | 331 | Scrub Oak | 153 | Black bear, gray fox |
| K_F23_112211 | MHW | MHC | 16 | 1133 | 100% | 16 | 148 | Scrub Oak | 41 | Bobcat, California ground squirrel, fisher, western spotted skunk |
| K_M33_112211 | DFR | MHC | 6 | 989 | 100% | 9 | 288 | Douglas Fir | 142 | Coyote, gray fox, puma, white-footed woodrat |
| K_F23_113011 | CRC | AG | 12 | 1075 | 91% | 21 | 215 | Scrub Oak | 30 | Black bear |
| K_M33_121511 | MHC | DFR | 50 | 1168 | 97% | 12 | 267 | Douglas Fir | 48 | Black bear, deer mouse |
| K_M33_121811 | MHC | DFR | 50 | 1164 | 97% | 12 | 267 | Douglas Fir | 62 | Black bear |
| K_F17_123111 | DFR | MHC | 8 | 938 | 97% | 5 | 261 | Douglas Fir | 175 | Common raven, fisher, gray fox |
| K_F17_010412 | KMC | MHW | 12 | 1708 | 81% | 19 | 151 | Ponderosa Pine | 1 | Common raven |
| K_F19_011612 | MCH | MHC | 30 | 816 | 100% | 2 | 50 | Scrub Oak | 139 | gray fox, ringtail, scrub jay, white-footed woodrat |
| K_F17_012412 | KMC | MHC | 13 | 1244 | 99% | 7 | 308 | Douglas Fir | 4 | Common raven |
| K_F17_020612 | MHC | AG | 16 | 966 | 99% | 3 | 208 | Douglas Fir | 7 | Bobcat, coyote, deer mouse, gray fox, white-footed woodrat |
| K_F19_020912 | MHW | MHC | 45 | 614 | 89% | 7 | 69 | Live Oak | 0 | Scrub jay, Steller's jay |
| K_M33_022512 | MCH | MHW | 5 | 900 | 98% | 12 | 137 | Scrub Oak | 150 | Coyote, gray fox, ringtail |
| K_F17_022912 | MHW | AG | 25 | 710 | 80% | 40 | 300 | Big Leaf Maple | 354 | Black bear, deer mouse, ringtail |
| K_M33_031412 | MCP | KMC | 85 | 1269 | 94% | 4 | 142 | Manzanita | 497 | Black bear, bobcat, common raven, coyote, fisher, gray fox, gray squirrel, scrub jay, Steller's jay, western spotted skunk |
| K_F17_032212 | DFR | MHC | 10 | 1091 | 99% | 2 | 67 | Douglas Fir | 82 | Black bear, fisher, gray fox |
| K_M33_032212 | MHC | KMC | 60 | 1160 | 99% | 10 | 288 | Live Oak | 22 | Common raven, fisher, gray fox |
| K_M33_041412 | MCP | DFR | 5 | 1071 | 91% | 14 | 241 | Manzanita | 17 | Black bear, common raven, domestic dog, fisher, gray fox |
| K_F43_042412 | KMC | MHC | 9 | 1063 | 100% | 8 | 192 | Douglas Fir | 710 | Black bear, coyote, turkey vulture |
| K_F43_051012 | MHW | MHC | 9 | 976 | 100% | 5 | 262 | Scrub Oak | 55 | Black bear |
| K_F17_051112 | MCP | MHC | 6 | 1491 | 72% | 12 | 219 | Ceanothus | 33 | Black bear, gray fox |
| K_M33_051812 | KMC | AG | 12 | 1397 | 100% | 3 | 241 | Douglas Fir | 154 | Black bear, coyote |
| K_M33_061212 | KMC | MCP | 3 | 1754 | 100% | 2 | 158 | Incense Cedar | 133 | Black bear |
| K_F43_062012 | MHC | KMC | 7 | 1282 | 99% | 9 | 121 | Ponderosa Pine | 121 | Black bear, gray fox, turkey vulture |
| K_F43_062312 | MHC | MHW | 8 | 1512 | 97% | 24 | 164 | Douglas Fir | 284 | Black bear, turkey vulture |
| K_M33_062412 | KMC | MHC | 16 | 1487 | 100% | 3 | 120 | Douglas Fir | 81 | Black bear, turkey vulture |
| K_F43_062812 | RFR | KMC | 4 | 1886 | 96% | 14 | 330 | Red Fir | 96 | Black bear, turkey vulture |
| K_M33_070612 | MCP | KMC | 4 | 1565 | 93% | 12 | 87 | Chamise | 305 | Black bear, California ground squirrel, turkey vulture |
| K_F43_071712 | KMC | MCP | 3 | 1853 | 69% | 23 | 307 | Incense Cedar | 384 | Black bear, bobcat, coyote, turkey vulture |
| K_F43_072812 | KMC | MCP | 3 | 1790 | 97% | 10 | 40 | White Fir | 349 | Black bear, common raven, Douglas squirrel, golden-mantled ground squirrel, turkey vulture, white-footed woodrat |
| K_F43_080912 | BAR | KMC | 3 | 1985 | 24% | 22 | 122 | Incense Cedar | 155 | Black bear, California ground squirrel, coyote, golden-mantled ground squirrel, turkey vulture |
| K_F43_081812 | MRI | WTR | 2 | 861 | 92% | 4 | 321 | Alder | 73 | Black bear, California ground squirrel, fisher, gray fox, raccoon, ringtail, western spotted skunk |
| K_F43_091312 | DFR | KMC | 3 | 1652 | 100% | 3 | 123 | Douglas Fir | 178 | Black bear, white-footed woodrat |
| K_M33_091412 | MRI | WTM | 4 | 1808 | 100% | 2 | 187 | Willow | 3 | Coyote |
| K_F43_092712 | DFR | KMC | 3 | 1630 | 98% | 8 | 19 | Douglas Fir | 54 | Black bear, coyote |
| K_F43_100712 | MHW | MHC | 12 | 1451 | 91% | 21 | 186 | Black Oak | 256 | Black bear, California ground squirrel, Douglas squirrel, fisher, gray fox, ringtail, Steller's jay, western spotted skunk, white-footed woodrat |
| K_F43_101312 | MHC | DFR | 12 | 1171 | 99% | 9 | 278 | Black Oak | 183 | Black bear, common raven, coyote, gray fox |
| K_F43_101812 | MRI | MHW | 20 | 737 | 100% | 24 | 250 | Alder | 244 | Black bear, California ground squirrel, gray fox, ringtail |

A2

| Paired Control Carcass Name | Primary Habitat | Secondary Habitat | Distance (meters) | Elevation (meters) | Canopy Cover | Slope | Aspect | Overhead Tree | Sum Feeding Time | Scavengers Present |
| --- | --- | --- | --- | --- | --- | --- | --- | --- | --- | --- |
| P_F1_052011 | MCH | MHC | 20 | 1283 | 100% | 26 | 82 | Scrub Oak | 168 | Black bear, coyote, western spotted skunk, white-footed woodrat |
| P_M36_052211 | MHW | BAR | 14 | 483 | 100% | 4 | 32 | Live Oak | 215 | Common raven, turkey vulture |
| P_M36_052611 | MHW | AG | 8 | 461 | 100% | 11 | 296 | California Buckeye | 775 | Common raven, domestic dog, turkey vulture |
| P_M36_060511 | DFR | KMC | 18 | 1575 | 100% | 14 | 42 | Douglas Fir | 1081 | Black bear, California ground squirrel, coyote, |
| P_M36_061111 | MHW | KMC | 12 | 1378 | 100% | 22 | 302 | Live Oak | 474 | Black bear, bobcat, gray fox |
| P_F1_062111 | KMC | MHC | 15 | 1497 | 85% | 19 | 72 | Red Fir | 73 | Black bear |
| P_F1_063011 | MHC | MCP | 3 | 1520 | 100% | 6 | 243 | Black Oak | 195 | Black bear, bobcat, gray fox, turkey vulture |
| P_F1_070311 | MHC | MCP | 12 | 756 | 98% | 8 | 160 | Black Oak | 41 | Black bear, coyote |
| P_F17_082811 | KMC | DFR | 10 | 1333 | 93% | 12 | 11 | Ponderosa Pine | 40 | Black bear, California ground squirrel, turkey vulture |
| P_M33_091711 | MHC | KMC | 2 | 1266 | 100% | 8 | 194 | Douglas Fir | 40 | Black bear, California ground squirrel, gray fox |
| P_F23_091711 | KMC | MHC | 15 | 1334 | 100% | 19 | 348 | Incense Cedar | 711 | Black bear, California ground squirrel, coyote, |
| P_F17_100311 | MHC | KMC | 2 | 1594 | 99% | 18 | 89 | Ponderosa Pine | 0 | California ground squirrel, chipmunk, Douglas squirrel |
| P_M33_100911 | KMC | WFR | 12 | 1674 | 99% | 12 | 59 | Ponderosa Pine | 71 | Coyote, fisher |
| P_F19_101611 | MHW | MCH | 10 | 983 | 96% | 20 | 334 | Brewer's Oak | 279 | Black bear, common raven, coyote, gray fox, western spotted skunk |
| P_F17_102011 | MHC | AG | 25 | 1468 | 91% | 6 | 260 | Black Oak | 105 | Black bear, California ground squirrel, coyote, deer mouse, western spotted skunk, white-footed woodrat |
| P_F19_102511 | MCH | MHW | 3 | 1306 | 100% | 17 | 93 | Manzanita | 438 | Black bear, gray fox, western spotted skunk |
| P_F23_102811 | MHW | MHC | 6 | 1039 | 100% | 11 | 164 | Live Oak | 213 | Black bear, domestic dog, western spotted skunk |
| P_F19_110611 | MCH | AG | 7 | 1080 | 99% | 22 | 99 | Scrub Oak | 9 | California ground squirrel, domestic dog, red-tailed hawk |
| P_F17_110911 | MHW | MCP | 3 | 1034 | 92% | 19 | 179 | Black Oak | 240 | Domestic dog, gray fox, raccoon, western spotted skunk |
| P_F23_111111 | MRI | BOP | 5 | 540 | 93% | 16 | 25 | Dogwood | 270 | Black bear, coyote, gray fox, striped skunk, western spotted skunk |
| P_F17_112011 | MHC | MHW | 14 | 1002 | 97% | 15 | 303 | Scrub Oak | 37 | coyote, deer mouse, fisher, gray fox |
| P_F23_112211 | MHW | MHC | 3 | 1328 | 100% | 18 | 108 | Scrub Oak | 314 | Black bear, bobcat, common raven, fisher |
| P_M33_112211 | DFR | MHC | 7 | 1071 | 100% | 8 | 324 | Douglas Fir | 270 | common raven, deer mouse, gray fox, white-footed woodrat |
| P_F23_113011 | MCH | AG | 6 | 964 | 98% | 14 | 276 | Scrub Oak | 338 | Black bear, gray fox |
| P_M33_121511 | MHC | DFR | 10 | 1206 | 96% | 4 | 71 | Douglas Fir | 205 | Deer mouse, fisher, gray fox, raccoon |
| P_M33_121811 | MHC | DFR | 40 | 1175 | 95% | 5 | 100 | Douglas Fir | 38 | Gray fox |
| P_F17_123111 | DFR | MHC | 12 | 1435 | 98% | 7 | 97 | Douglas Fir | 342 | Bobcat, coyote |
| P_F17_010412 | KMC | MHW | 9 | 1602 | 84% | 16 | 112 | Ponderosa Pine | 9 | Fisher, Steller's jay |
| P_F19_011612 | MHW | MHC | 20 | 1140 | 100% | 20 | 97 | Scrub Oak | 335 | Fisher, gray fox, scrub jay, white-footed woodrat |
| P_F17_012412 | KMC | MHC | 50 | 1043 | 98% | 10 | 131 | Douglas Fir | 258 | Bobcat, domestic dog, gray fox, western spotted skunk |
| P_F17_020612 | MHC | AG | 16 | 898 | 98% | 9 | 100 | Douglas Fir | 549 | Bobcat, coyote, gray fox, puma, ringtail |
| P_F19_020912 | MHW | MHC | 35 | 1017 | 92% | 11 | 147 | Live Oak | 372 | Fisher, gray fox |
| P_M33_022512 | MCH | MHW | 5 | 1245 | 98% | 18 | 157 | Live Oak | 113 | American Robin, gray fox, puma, Steller's jay, white-footed woodrat |
| P_F17_022912 | MHW | AG | 6 | 949 | 89% | 21 | 332 | Big Leaf Maple | 53 | Black bear, bobcat, coyote, gray fox |
| P_M33_031412 | MCP | KMC | 25 | 1273 | 94% | 2 | 253 | Manzanita | 649 | Black bear, bobcat, common raven, deer mouse, fisher, gray fox, gray squirrel, Steller's jay, white-footed woodrat |
| P_F17_032212 | DFR | MHC | 12 | 1255 | 99% | 4 | 9 | Douglas Fir | 634 | Black bear, common raven, coyote, turkey vulture |
| P_M33_032212 | MHC | KMC | 40 | 1044 | 99% | 8 | 214 | Live Oak | 358 | Common raven, domestic dog, gray fox, puma, ringtail |
| P_M33_041412 | MCP | DFR | 3 | 875 | 88% | 16 | 229 | Manzanita | 213 | Common raven, domestic dog, gray fox, turkey vulture |
| P_F43_042412 | KMC | MHC | 8 | 1162 | 100% | 5 | 221 | Douglas Fir | 102 | Black bear, common raven, fisher, turkey vulture |
| P_F43_051012 | MHW | MHC | 6 | 971 | 100% | 7 | 296 | Scrub Oak | 671 | Black bear, common raven, fisher, gray fox, striped skunk, turkey vulture |
| P_F17_051112 | MCP | MHC | 4 | 1986 | 62% | 9 | 244 | Ceanothus | 86 | Back bear, common raven, coyote, golden eagle |
| P_M33_051812 | KMC | AG | 5 | 1218 | 100% | 4 | 291 | Douglas Fir | 348 | Black bear, coyote, gray fox |
| P_M33_061212 | KMC | MCP | 4 | 1621 | 98% | 2 | 182 | Incense Cedar | 218 | Black bear |
| P_F43_062012 | MHC | KMC | 15 | 1194 | 93% | 7 | 236 | Ponderosa Pine | 42 | Coyote, puma, turkey vulture |
| P_F43_062312 | MHC | MHW | 14 | 1203 | 98% | 8 | 330 | Douglas Fir | 217 | Black bear, California ground squirrel, coyote, deer mouse, gray fox |
| P_M33_062412 | KMC | MHC | 18 | 1743 | 100% | 14 | 86 | Douglas Fir | 38 | Black bear, white-footed woodrat |
| P_F43_062812 | RFR | KMC | 2 | 1831 | 97% | 18 | 308 | Red Fir | 115 | Black bear, coyote, deer mouse, turkey vulture |
| P_M33_070612 | MCP | KMC | 5 | 1743 | 89% | 5 | 51 | Chamise | 55 | Black bear |
| P_F43_071712 | KMC | MCP | 2 | 1783 | 72% | 6 | 93 | Incense Cedar | 234 | Common raven, turkey vulture |
| P_F43_072812 | KMC | MCP | 8 | 1790 | 97% | 10 | 100 | White Fir | 108 | Black bear, chipmunk, common raven, turkey vulture |
| P_F43_080912 | BAR | KMC | 4 | 1950 | 20% | 23 | 258 | Incense Cedar | 659 | Black bear, California ground squirrel, common raven, coyote, turkey vulture |
| P_F43_081812 | MRI | WTR | 4 | 1116 | 95% | 3 | 306 | Alder | 71 | Black bear, coyote, fisher, gray fox, puma, white-footed woodrat |
| P_F43_091312 | DFR | KMC | 6 | 1682 | 100% | 4 | 82 | Douglas Fir | 94 | Black bear, fisher |
| P_M33_091412 | MRI | WTM | 2 | 1861 | 100% | 6 | 93 | Willow | 94 | Black bear, common raven |
| P_F43_092712 | DFR | KMC | 5 | 1537 | 99% | 14 | 71 | Douglas Fir | 16 | Black bear, bobcat, domestic dog, golden-mantled ground squirrel |
| P_F43_100712 | MHW | MHC | 6 | 1377 | 97% | 23 | 205 | Black Oak | 138 | Black bear, Douglas squirrel, gray fox, ringtail, Steller's jay |
| P_F43_101312 | MHC | DFR | 5 | 1141 | 99% | 8 | 322 | Black Oak | 254 | Black bear, coyote, fisher, gray fox |
| P_F43_101812 | MRI | MHW | 18 | 1121 | 100% | 13 | 292 | Alder | 202 | Gray fox, ringtail |
