# Supplementary material for: Trophic Facilitation or Limitation? Comparative Effects of Pumas and Black Bears on the Scavenger Community
Source: PLoS One. 2014 Jul 10;9(7):e102257. doi: 10.1371/journal.pone.0102257 (PMC4092109; doi:10.1371/journal.pone.0102257)
Supplement: Appendix S2 — The habitat characteristics, sum feeding time, and scavengers present at the black bear experimental carcasses. (DOCX) [file pone.0102257.s002.docx]

Appendix B. The habitat characteristics, sum feeding time, and scavengers present at the black bear experimental carcasses. Habitat characteristics reported include the primary and secondary habitat types [26], the distance to the secondary habitat, the elevation, canopy cover, slope, aspect, and overhead tree species. The scavenger sum feeding times (excluding rodents and small birds), and each of the scavengers that occurred at the carcass are also reported.

| Name | Carcass Type | Date Set Out | Habitat | Secondary Habitat | Distance (meters) | Elevation (meters) | Overhead Tree Species | Canopy Cover | Slope | Aspect | Sum Feeding Time | Species |  |
| --- | --- | --- | --- | --- | --- | --- | --- | --- | --- | --- | --- | --- | --- |
| B010810-1 | Absent | 1/8/2010 | BOW | AG | 8 | 1045 | Blue Oak | 81% | 8 | 15 | 20 | Fisher, gray fox |  |
| B013110-1 | Absent | 1/31/2010 | MCP | AG | 1 | 874 | Manzanita | 16% | 12 | 183 | 2 | Common raven |  |
| B013110-2 | Absent | 1/31/2010 | BOW | AG | 4 | 845 | Scrub Oak | 95% | 12 | 39 | 445 | Gray fox |  |
| B013110-3 | Excluded | 1/31/2010 | MHW | WTR | 6 | 883 | Mountain Mahogany | 84% | 1 | 143 | - | Common raven, golden eagle, gray fox |  |
| B013110-4 | Excluded | 1/31/2010 | BOP | AG | 2 | 851 | Gray Pine | 89% | 22 | 172 | - | Coyote, gray fox |  |
| B020410-1 | Absent | 2/4/2010 | MHC | WTR | 12 | 774 | Douglas Fir | 100% | 24 | 137 | 22 | Bobcat, gray fox |  |
| B020610-1 | Absent | 2/6/2010 | MCP | PPN | 18 | 833 | Manzanita | 93% | 11 | 101 | 98 | Bobcat, common raven, coyote, gray fox |  |
| B022110-1 | Absent | 2/21/2010 | PPN | AG | 8 | 1323 | Ponderosa Pine | 73% | 2 | 342 | 639 | Bobcat, common raven, fisher, gray fox |  |
| B022110-2 | Absent | 2/21/2010 | KMC | AG | 2 | 1400 | Black Oak | 15% | 6 | 53 | 31 | Common raven |  |
| B022710-1 | Absent | 2/27/2010 | MHW | AG | 30 | 1200 | Live Oak | 100% | 31 | 87 | 148 | Bobcat, coyote, domestic dog, fisher, spotted skunk |  |
| B030210-1 | Excluded | 3/2/2010 | MCH | AG | 26 | 963 | Scrub Oak | 97% | 21 | 271 | 26 | Bobcat, common raven, gray fox |  |
| B030210-2 | Absent | 3/2/2010 | MHW | AG | 21 | 1147 | Live Oak | 95% | 25 | 216 | 248 | Bobcat, California ground squirrel, coyote, domestic dog, gray fox, raccoon, Steller's jay, white-footed woodrat |  |
| B032910-1 | Absent | 3/29/2010 | MHW | AG | 12 | 1198 | Live Oak | 100% | 18 | 74 | 123 | Bobcat, gray fox |  |
| B033010-1 | Absent | 3/30/2010 | MHC | AG | 1 | 1537 | Brewers Oak | 57% | 9 | 266 | 16 | Common raven, gray fox, white-footed woodrat |  |
| B033010-2 | Absent | 3/30/2010 | PPN | AG | 8 | 1825 | Ponderosa Pine | 86% | 7 | 2 | 255 | Bobcat, Steller's jay |  |
| B033010-3 | Present | 3/30/2010 | MHC | MHW | 12 | 1219 | Ponderosa Pine | 78% | 5 | 210 | 11 | Fisher |  |
| B040710-1 | Present | 4/7/2010 | MHW | AG | 22 | 1122 | Black Oak | 99% | 3 | 140 | 75 | Common raven, deer mouse, fisher, kangaroo rat, turkey vulture, white-footed woodrat |  |
| B041010A | Absent | 4/10/2010 | MHC | MHW | 12 | 1195 | Douglas Fir | 0.97 | 9 | 266 | 107 | Fisher, turkey vulture |  |
| B041610-1 | Excluded | 4/16/2010 | MHC | MCH | 8 | 1164 | Live Oak | 95% | 18 | 220 | - | Common raven, gray fox, turkey vulture |  |
| B041610-2 | Absent | 4/16/2010 | AG | BOW | 6 | 1290 | Ponderosa Pine | 61% | 16 | 38 | 783 | Bobcat, California ground squirrel, common raven, coyote, fisher, golden eagle |  |
| B041610-3 | Absent | 4/16/2010 | PPN | MHC | 9 | 1252 | Ponderosa Pine | 100% | 4 | 246 | 439 | Bobcat, common raven, coyote, fisher, turkey vulture, white-footed woodrat |  |
| B041910-1 | Absent | 4/19/2010 | MHC | AG | 13 | 1194 | Ponderosa Pine | 98% | 17 | 302 | 104 | Common raven, |  |
| B050110-1 | Present | 5/1/2010 | MHC | AG | 24 | 1202 | Live Oak | 97% | 2 | 171 | 84 | Common raven, gray fox, gray squirrel, turkey vulture |  |
| B050410-1 | Excluded | 5/4/2010 | AG | MHW | 9 | 1106 | Black Oak | 44% | 17 | 328 | - |  |  |
| B052710-1 | Present | 5/27/2010 | KMC | AG | 1 | 1375 | Ponderosa Pine | 43% | 19 | 294 | 29 | Gray fox |  |
| B052710-2 | Present | 5/27/2010 | MHW | MHC | 7 | 917 | Manzanita | 64% | 19 | 324 | 226 | Turkey vulture |  |
| B052710-3 | Present | 5/27/2010 | MHC | AG | 12 | 1191 | Live Oak | 98% | 3 | 304 | 11 | Common raven, fisher, golden eagle, turkey vulture |  |
| B080910-1 | Excluded | 8/9/2010 | DFR | WTM | 6 | 1589 | Douglas Fir | 100% | 1 | 62 | - |  |  |
| B102210-1 | Present | 10/22/2010 | MHC | MCP | 8 | 1354 | Live Oak | 99% | 28 | 64 | 1 | Coyote, gray squirrel |  |
| B102210-2 | Present | 10/22/2010 | MHC | DFR | 6 | 1242 | Scrub Oak | 100% | 10 | 276 | 411 | Common raven, fisher, gray fox, red-tailed hawk |  |
| B102910-1 | Present | 10/29/2010 | KMC | MCH | 16 | 1514 | Douglas Fir | 99% | 19 | 61 | 241 | California ground squirrel, common raven, coyote, fisher, red-tailed hawk, ringtail |  |
| B011411-1 | Absent | 1/14/2011 | MCH | KMC | 10 | 954 | Manzanita | 100% | 11 | 316 | 348 | Common raven, coyote, domestic dog, gray fox, puma |  |
| B020511-1 | Absent | 2/5/2011 | KMC | PPN | 10 | 1237 |  | 100% | 15 | 20 | 1065 | Coyote, fisher, gray fox, golden eagle |  |
| B020911-1 | Present | 2/9/2011 | KMC | AG | 5 | 1339 | White Fir | 100% | 12 | 188 | 392 | American Crow, common raven, coyote, fisher, gray squirrel, Steller's jay |  |
| B022611-1 | Absent | 2/26/2011 | MHC | MCH | 10 | 1226 | Black Oak | 95% | 14 | 41 | 150 | Bobcat, coyote, fisher, gray fox |  |
| B031411-1 | Present | 3/14/2011 | MHW | KMC | 14 | 907 | Live Oak | 100% | 19 | 344 | 14 | Common raven, domestic dog, golden eagle, gray squirrel |  |
| B031611-1 | Present | 3/16/2011 | MCH | AG | 3 | 862 | Manzanita | 26% | 2 | 301 | 35 | Coyote, golden eagle, gray squirrel |  |
| B040311-1 | Absent | 4/3/2011 | PPN | AG | 4 | 1299 | Ponderosa Pine | 92% | 5 | 304 | 231 | Common raven, gray fox, puma, turkey vulture |  |
| B040311-2 | Absent | 4/3/2011 | MHC | MCH | 6 | 925 | Black Oak | 99% | 9 | 283 | 539 | Turkey vulture |  |
| B040511-1 | Absent | 4/5/2011 | DFR | BAR | 3 | 1192 | Douglas Fir | 97% | 26 | 25 | 1642 | Fisher, gray fox, turkey vulture |  |
| B040611-1 | Absent | 4/6/2011 | MHW | AG | 14 | 1178 | Manzanita | 97% | 5 | 28 | 99 | Common raven, gray fox, puma, turkey vulture |  |
| B041011-1 | Absent | 4/10/2011 | MCH | MHW | 9 | 976 | Manzanita | 99% | 3 | 164 | 484 | Bobcat, domestic dog, turkey vulture |  |
| B041111-1 | Excluded | 4/11/2011 | MCH | KMC | 5 | 1261 | Manzanita | | 2 | 341 | - | Gray fox | |
| B041911-1 | Excluded | 4/19/2011 | MHW | BOW | 15 | 1049 | Scrub Oak | 96% | 10 | 14 | - | Golden eagle, turkey vulture |  |
| B042211-1 | Present | 4/22/2011 | MHW | AG | 1 | 1001 | White Oak | 86% | 5 | 95 | 69 | American crow, bald eagle, coyote, golden eagle, turkey vulture, |  |
| B042611-1 | Present | 4/26/2011 | PPN | AG | 4 | 1299 | Ponderosa Pine | 92% | 5 | 304 | 0 |  |  |
| B060611-1 | Present | 6/6/2011 | DFR | MHW | 9 | 912 | Douglas Fir | 100% | 12 | 352 | 165 | Gray squirrel, turkey vulture |  |
| B060611-2 | Present | 6/6/2011 | DFR | MHW | 14 | 879 | Douglas Fir | 95% | 4 | 318 | 0 |  |  |
| B060611-3 | Present | 6/6/2011 | KMC | MHC | 18 | 1221 | Ponderosa Pine | 98% | 3 | 194 | 1 | Turkey vulture |  |
| B060811-1 | Present | 6/8/2011 | PPN | MHW |  | 1423 | Ponderosa Pine | 99% | 4 | 206 | 21 | Bobcat |  |
| B070711-1 | Present | 7/7/2011 | MHC | AG | 6 | 967 | Black Oak | 100% | 10 | 38 | 5 | Common raven, turkey vulture |  |
| B071111-1 | Present | 7/11/2011 | PPN | AG | 8 | 1402 | Ponderosa Pine | 99% | 6 | 347 | 75 | Common raven, coyote, turkey vulture |  |
| B111311-1 | Present | 11/13/2011 | MHC | KMC | 8 | 1254 | Blue Oak | 76% | 2 | 214 | 4 | Gray fox |  |
| B111611-1 | Absent | 11/16/2011 | MHC | DFR | 17 | 1172 | Douglas Fir | 99% | 6 | 223 | 245 | Common raven, domestic dog, gray fox, ringtail, spotted skunk |  |
| B112111-1 | Present | 11/21/2011 | MHC | MCP | 8 | 1259 | Ponderosa Pine | 95% | 9 | 279 | 2 | Common raven |  |
| B122111-1 | Absent | 12/21/2011 | PPN | MHC | 25 | 1369 | Ponderosa Pine | 88% | 7 | 161 | 103 | Coyote, gray fox |  |
| B123111-1 | Absent | 12/31/2011 | MHW | MHC | 4 | 1160 | Live Oak | 100% | 12 | 296 | 33 | Coyote, domestic dog, fisher, gray fox, puma |  |
| B010112-1 | Absent | 1/1/2012 | KMC | MRI | 6 | 1327 | Douglas Fir | 93% | 6 | 181 | 439 | Common raven, coyote, golden eagle, puma |  |
| B010112-2 | Absent | 1/1/2012 | WFR | KMC | 12 | 1435 | White Fir | 98 | 7 | 97 | 342 |  |  |
| B011112-1 | Absent | 1/11/2012 | MHC | KMC | 50 | 965 | Manzanita | 58% | 7 | 301 | 504 | Gray fox, great horned owl, puma, spotted skunk |  |
| B012112-1 | Absent | 1/21/2012 | MHW | AG | 12 | 812 | Madrone | 99% | 10 | 158 | 632 | Golden eagle, gray fox, spotted skunk |  |
| B012112-2 | Absent | 1/21/2012 | DFR | MHC | 8 | 924 | Douglas Fir | 99% | 4 | 332 | 821 | Common raven, coyote, fisher, golden eagle, gray fox |  |
| B012412-1 | Absent | 1/24/2012 | KMC | MHC | 17 | 1340 | Doug Fir | 98% | 2 | 260 | 112 | Coyote |  |
| B012712-1 | Absent | 1/27/2012 | MHC | KMC | 20 | 1258 | Live Oak | 88% | 9 | 304 | 454 | Gray fox |  |
| B020512-1 | Absent | 2/5/2012 | MHC | KMC | 50 | 965 | Manzanita | 58% | 7 | 301 | 155 | Coyote, gray fox, gray squirrel, spotted skunk |  |
| B022112-1 | Absent | 2/22/2012 | MHC | KMC | 50 | 951 | Manzanita | 82% | 6 | 301 | 471 | Bobcat, common raven, coyote, golden eagle, gray fox, |  |
| B022112-2 | Absent | 2/22/2012 | KMC | MHC | 25 | 949 | Black Oak | 97% | 5 | 248 | 97 | Fisher, northern goshawk |  |
| B022612-1 | Absent | 2/26/2012 | KMC | MHC | 25 | 971 | Douglas Fir | 93% | 4 | 230 | 489 | Bobcat, coyote |  |
| B022812-2 | Absent | 2/28/2012 | MHW | AG | 35 | 864 | Manzanita | 94% | 5 | 157 | 1025 | Gray fox, turkey vulture |  |
| B022912-1 | Excluded | 2/29/2012 | DFR | KMC | 70 | 1518 | Douglas Fir | 99% | 14 | 25 | - | Coyote, Dougals squirrel |  |
| B031112-1 | Absent | 3/11/2012 | MHC | MHW | 30 | 1494 | Ponderosa Pine | 0.02 | 0 | 348 | 24 | Common raven, fisher, golden eagle, Steller's jay |  |
| B031112-2 | Absent | 3/11/2012 | MHW | MHC | 10 | 1349 | Brewers Oak | 91% | 13 | 286 | 130 | Common raven, coyote, fisher |  |
| B031112-3 | Excluded | 3/11/2012 | KMC | MHC | 100 | 1424 | Douglas Fir | 99% | 19 | 131 | - | Bobcat, coyote, fisher |  |
| B031112-4 | Absent | 3/11/2012 | MHC | MHW | 35 | 1210 | Douglas Fir | 93% | 1 | 273 | 485 | Common raven, coyote, fisher, ringtail |  |
| B031112-5 | Absent | 3/11/2012 | MHC | KMC | 110 | 1359 | Deer Brush | 0.6 | 3 | 22 | 807 | Common raven, puma, Steller's jay |  |
| B032012-1 | Absent | 3/20/2012 | KMC | MHC | 110 | 1236 | Douglas Fir | 97% | 1 | 314 | 683 | Common raven, domestic dog, fisher, gray fox |  |
| B032812-1 | Absent | 3/28/2012 | DFR | MHC | 70 | 1286 | Douglas Fir | 99% | 2 | 265 | 868 | Bobcat, common raven, fisher, gray fox, ringtail |  |
| B032812-2 | Present | 3/28/2012 | MHC | KMC | 40 | 928 | Douglas Fir | 97% | 2 | 298 | 148 | Common raven, fisher, gray fox |  |
| B032812-3 | Present | 3/28/2012 | DFR | MHC | 90 | 974 | Douglas Fir | 99% | 3 | 330 | 79 | Common raven, gray fox, ringtail, white-footed woodrat |  |
| B032812-4 | Present | 3/28/2012 | MHC | DFR | 35 | 728 | Madrone | 94% | 10 | 273 | 103 | Common raven, coyote, domestic dog, gray fox, gray squirrel, ringtail, turkey vulture, white-footed woodrat |  |
| B041512-1 | Absent | 4/15/2012 | MCP | DFR | 3 | 875 | Manzanita | 88% | 16 | 229 | 213 | Fisher, puma |  |
| B042212-1 | Present | 4/22/2012 | DFR | MHC | 3 | 952 | Douglas Fir | 95% | 21 | 253 | 282 | Common raven, turkey vulture |  |
| B053112-1 | Present | 5/31/2012 | DFR | KMC | 11 | 1523 | Douglas Fir | 100% | 4 | 233 | 28 | Turkey vulture |  |
| B061012-1 | Present | 6/10/2012 | MRI | AG | 12 | 1194 | Big-leaf Maple | 98% | 14 | 272 | 15 | Turkey vulture |  |
| B061612-1 | Absent | 6/16/2012 | DFR | MHC | 3 | 962 | Douglas Fir | 100% | 12 | 263 | 108 |  |  |
| B061612-2 | Present | 6/16/2012 | BOW | AG | 12 | 809 | Blue Oak | 100% | 9 | 241 | 4 | Gray fox |  |
| B061612-3 | Present | 6/16/2012 | PPN | AG | 9 | 1312 | Ponderosa Pine | 100% | 7 | 132 | 212 | Common raven, turkey vulture |  |
| B062212-1 | Absent | 6/22/2012 | MHC | AG | 7 | 1078 | Black Oak | 97% | 12 | 352 | 330 | Domestic dog, gray fox, turkey vulture |  |
| B080412-1 | Present | 8/4/2012 | DFR | MHC | 5 | 1217 | Douglas Fir | 98% | 12 | 360 | 0 |  |  |
| B080612-1 | Present | 8/6/2012 | KMC | MCP | 2 | 1793 | Ponderosa Pine | 89% | 7 | 305 | 134 | Turkey vulture |  |
| B080612-2 | Present | 8/6/2012 | MHC | KMC | 15 | 1480 | Douglas Fir | 99% | 6 | 164 | 29 | Turkey vulture |  |
| B081512-1 | Present | 8/15/2012 | MHW | MCP | 13 | 1482 | Black Oak | 98% | 21 | 354 | 15 | Turkey vulture |  |
| B091512-2 | Present | 9/15/2012 | WFR | RFR | 2 | 1912 | White Fir | 87% | 9 | 85 | 1 | Common raven |  |
| B091612-1 | Present | 9/16/2012 | KMC | MRI | 10 | 1263 | Incense Cedar | 86% | 13 | 16 | 47 | Coyote, fisher, gray fox |  |
| B092412-1 | Present | 9/24/2012 | MHC | MCH | 7 | 1121 | Douglas Fir | 86% | 14 | 351 | 0 |  |  |
| B092412-2 | Present | 9/24/2012 | MHC | PPN | 7 | 1428 | Ponderosa Pine | 89% | 17 | 208 | 64 | Common raven, coyote, turkey vulture |  |
| B093012-1 | Present | 9/30/2012 | MHC | KMC | 10 | 1495 | Brewer's Oak | 89% | 7 | 127 | 1 | Bobcat |  |
| B100112-1 | Present | 10/1/2012 | MHC | MHW | 7 | 1390 | Brewer's Oak | 97% | 16 | 246 | 71 | Common raven, gray fox, striped skunk, turkey vulture |  |
| B100112-2 | Present | 10/1/2012 | KMC | MHC | 13 | 1495 | Ponderosa Pine | 82% | 2 | 124 | 1 | Puma, striped skunk |  |
| B100612-1 | Present | 10/6/2012 | MRI | AG | 2 | 1182 | Alder | 100% | 8 | 286 | 593 | Fisher, gray fox, spotted skunk |  |
